# Carbon Biogeochemistry of the Estuaries Adjoining the Indian Sundarbans Mangrove Ecosystem: A Review

**DOI:** 10.3390/life13040863

**Published:** 2023-03-23

**Authors:** Isha Das, Abhra Chanda, Anirban Akhand, Sugata Hazra

**Affiliations:** 1 School of Oceanographic Studies, Jadavpur University, Kolkata 700032, India; 2 Department of Ocean Science, Hong Kong University of Science and Technology, Kowloon, Hong Kong SAR, China

**Keywords:** air–water CH_4_ flux, air–water CO_2_ flux, dissolved inorganic carbon, dissolved organic carbon, partial pressure of CH_4_, partial pressure of CO_2_, particulate organic carbon, total alkalinity, salinity

## Abstract

The present study reviewed the carbon-biogeochemistry-related observations concerning CO_2_ and CH_4_ dynamics in the estuaries adjoining the Indian Sundarbans mangrove ecosystem. The review focused on the partial pressure of CO_2_ and CH_4_ [*p*CO_2(water)_ and *p*CH_4(water)_] and air–water CO_2_ and CH_4_ fluxes and their physical, biogeochemical, and hydrological drivers. The riverine-freshwater-rich Hooghly estuary has always exhibited higher CO_2_ emissions than the marine-water-dominated Sundarbans estuaries. The mangrove sediment porewater and recirculated groundwater were rich in *p*CO_2(water)_ and *p*CH_4(water)_, enhancing their load in the adjacent estuaries. Freshwater-seawater admixing, photosynthetically active radiation, primary productivity, and porewater/groundwater input were the principal factors that regulated *p*CO_2(water)_ and *p*CH_4(water)_ and their fluxes. Higher chlorophyll-*a* concentrations, indicating higher primary production, led to the furnishing of more organic substrates that underwent anaerobic degradation to produce CH_4_ in the water column. The northern Bay of Bengal seawater had a high carbonate buffering capacity that reduced the *p*CO_2(water)_ and water-to-air CO_2_ fluxes in the Sundarbans estuaries. Several authors traced the degradation of organic matter to DIC, mainly following the denitrification pathway (and pathways between aerobic respiration and carbonate dissolution). Overall, this review collated the significant findings on the carbon biogeochemistry of Sundarbans estuaries and discussed the areas that require attention in the future.

## 1. Introduction

The term “global warming” and its connection with greenhouse gases and ocean circulations came to the limelight in 1975 [1], and the scientific community observed a consistent increase in the global temperature to cross the baseline values since 1976 [2]. In this regard, the Earth Summit in 1992 realized the need for a global partnership for sustainable development, ensuring environmental protection and improved human life. Later, in 2015, the UN Sustainable Development Summit created 17 sustainable development goals (SDGs) to achieve a world with reduced effects from climate change (https://sdgs.un.org/goals, accessed on 30 September 2022). Since the advent of global warming and climate change, countless studies have tried to understand the interactions between climate change and the Earth’s environmental systems. Throughout the industrial period, greenhouse gas emissions have increased significantly, and the environment and oceans have received increased amounts of carbon, nitrogen, and phosphorous. This excess carbon and nutrients affected the coastal zones, which include tidal wetlands, estuaries, and continental shelf waters [3]. This anthropogenic loading changes the nutrient cycling and greenhouse gas emissions from coastal systems [4].

Estuaries are dynamic and responsive systems with considerable environmental variability as they bridge the terrestrial sector with the oceans [5]. They also play a significant role in global carbon cycling. As per recent studies, the net carbon dioxide (CO_2_) emission from the estuaries (0.1 to 0.4 Pg C (carbon) yr^−1^) is equivalent to one-quarter of the net uptake of atmospheric CO_2_ by the open ocean [6,7,8,9]. Besides calculating the global carbon budget, coastal carbon cycles also help comprehend several ecological and geochemical phenomena, as carbon is the common currency often used to describe them [10]. Coastal issues such as hypoxia due to oxidation of organic carbon [11], acidification resulting from anthropogenic and organic CO_2_ invasion [12], and the loss of blue carbon due to human development and sea level rise [13] require a thorough understanding of the coastal carbon dynamics. In the face of rapid coastal development and accelerated climate change, it is essential to understand the role of nearshore ecosystems as carbon sources and sinks through coastal carbon cycle studies [10].

Among the greenhouse gases driving climate change, CO_2_ and methane (CH_4_) are the primary ones. CO_2_ and CH_4_ contribute 73% and 19%, respectively, of the total greenhouse gases emitted globally [14]. Coal combustion (39%), oil combustion (31%), and natural gas combustion (18%) are the primary anthropogenic sources of CO_2_. In contrast, cattle (21%), rice cultivation (10%), natural gas production (14%), oil production (9%), landfilling (10%), wastewater processing (11%), and coal mining (10%) are the primary CH_4_ sources in the same category [14]. Though the percentage contribution of CH_4_ is lower than CO_2_ in the total greenhouse gases, methane’s global warming potential is 34 times stronger than CO_2_. Hence, a slight change in atmospheric concentration has a more significant warming effect [15]. CH_4_ is the second most important greenhouse gas after CO_2_, contributing almost 20% of global warming since the pre-industrial era [16]. Hence, understanding the dynamics of both CO_2_ and CH_4_ emissions will help predict future climatic changes. CO_2_ and CH_4_ fluxes refer to the exchange rate of carbon dioxide and methane per unit area per unit time between different carbon reservoirs within the ecosphere through physicochemical, biological, or anthropogenic activities. Mangroves absorb CO_2_ from the immediate environment and convert it into biomass through photosynthetic activity. Depending on the biogeochemical processes, the stored CO_2_ may have different fates [17]. Primary consumers may directly or indirectly consume the biomass or deposit it deep into the sediments. The carbon in the biomass may be re-mineralized and emitted as CO_2_ or exported as dissolved inorganic carbon (DIC). The carbon in the biomass may also undergo export into an adjacent ecosystem as dissolved and particulate organic carbon (DOC and POC). These can either be precipitated in sediments or consumed by aquatic organisms. 

The sources of CH_4_ emissions can be of three types: biogenic, thermogenic, and pyrogenic [15]. Biogenic sources include methanogenesis in wetlands, rice paddies, oxygen-poor reservoirs, the digestive systems of ruminants and termites, and waste deposit sites. Thermogenic CH_4_ forms over thousands of years through geological processes. CH_4_ comes into the open environment from the subsurface through natural features (terrestrial and marine seeps and mud volcanoes) and due to the exploitation of fossil fuels (coal, oil, natural gas, etc.). The pyrogenic sources refer to the incomplete combustion of biomass, soil carbon, biofuels, and fossil fuels [15]. Relatively fewer processes remove the CH_4_ released in the atmosphere. The primary sink of atmospheric CH_4_ is through chemical degradation, i.e., oxidation of CH_4_ by hydroxyl radicals (OH) in the troposphere, which accounts for 90% of the total global CH_4_ sink. The rest of the CH_4_ sinks are methanotrophic bacteria in the aerated soil [18,19], reactions with chlorine and atomic oxygen radicals in the stratosphere [20], and responses in the marine boundary layers [21].

Mangroves are among the most carbon-rich ecosystems on Earth [22,23], sequestering a large amount of carbon from the environment [24]. They are considered highly productive and carbon-dense ecosystems [25,26,27]. Globally, the rate of net primary production by the mangroves is 218 ± 72 Tg C yr^−1^ [28,29], approximately 1% of the carbon sequestration by the world’s forests [30]. Despite their limited distribution, mangroves play an enormous role in carbon sequestration with their high belowground root production and the ability to accumulate a large amount of organic matter in their substrates [31,32]. Hence, in the face of climate change and increased global CO_2_ and CH_4_ concentrations, the characterization of mangrove forests as carbon sources and sinks has gained added relevance [33]. Accurate quantification of the carbon pools and fluxes is crucial in developing mitigation strategies for reducing emissions from deforestation and forest degradation (REDD+) [34]. Several factors govern the net source or sink characteristics of a mangrove ecosystem. Transformation of the particulate and dissolved matter primarily creates a heterotrophic environment, eventually causing carbon emissions from the system [35,36,37,38,39]. However, net autotrophy in the New River Estuary of North Carolina, USA [40], and the estuaries on the southeast coast of Australia (viz. the Hastings River estuary, Camden Haven estuary, and Wallis Lake estuary) [41], caused these estuarine systems to behave as a net CO_2_ sink. Besides the biogeochemical characteristics such as trophic status [42] and net community production [43], the nature of geophysical processes in an estuary can also govern the CO_2_ flux. These include tidal amplitude [44,45], freshwater inflow [46,47], water residence time [48], connectivity of the system with salt marshes or tidal flats [8], and vertical stratification [49]. On the other hand, inundated wetlands rich in organic carbon tend to release a certain amount of CH_4_ into the surrounding environment. The emission rate depends on several climatic factors [50,51,52] along with some human-induced factors impacting methanogens and methanotrophs in the ecosystem [53,54,55]. Wetlands are the largest natural source of CH_4_, contributing about a third of the total global emissions (164 Tg yr^−1^) [50]. Soil temperature, water table, and the type of vegetation in the system often control the CH_4_ emissions from wetlands. Moreover, these relations vary across the wetland type (bog or swamp), the location of the wetland (temperate or tropical), and the level of disturbances the wetland experiences [51].

The Sundarbans mangrove ecosystem has always been a focal area of research in the recent past, considering the importance of mangrove forests in carbon sequestration discussed above. Overall, the CO_2_ and CH_4_ fluxes in the Indian Sundarbans come under the purview of three compartments, depending on the involvement of the carbon reservoirs and the direction of flux. They are: (i) atmosphere-biosphere flux (exchange of CO_2_ and CH_4_ between the mangrove forest and the atmosphere, i.e., above the forest canopy), (ii) soil-atmosphere flux (exchange of CO_2_ and CH_4_ between the exposed soil and the air below the canopy), and (iii) air–water flux (CO_2_ and CH_4_ exchange at the interface between the mangrove surrounding estuarine water and the atmosphere). Among these three types of fluxes, the present article reviewed the spatial and temporal dynamics of the air–water fluxes and the related carbon biogeochemistry of the estuarine water across the entire Sundarbans delta (Indian part).

Among the three types of carbon fluxes mentioned above, the air–water exchange is the most complex phenomenon as it involves several carbon parameters and their interplay with varied physicochemical, biogeochemical, and hydrological parameters. Unlike the mangrove canopy and the sediment surface, the aquatic column of the mangrove-adjacent estuaries furnishes a platform where several biogeochemical reactions coincide. Processes such as autotrophic utilization of aqueous CO_2_, degradation of POC and DOC, and the buffering capacity arising from the total alkalinity (TAlk) by DIC ratio can regulate the partial pressure of CO_2_ (*p*CO_2_) in water, which in turn governs the fluxes. Similarly, biochemical reactions carried out by methanogens and methanotrophs coupled with oxidation–reduction states of the water column control the partial pressure of CH_4_ (*p*CH_4_) in water and their fluxes. Aquatic carbon biogeochemistry has received substantially greater scientific attention in the Sundarbans estuaries owing to the intrinsic complexities of these concurrent mechanisms mentioned above. Many scholarly articles have come up in the past two decades in this regard to address multiple issues and research questions. Hence, we found it necessary to collate all this information acquired so far and synthesize the findings related to the carbon biogeochemistry of Sundarbans mangrove-dominated estuarine water, emphasizing the air–water CO_2_ and CH_4_ fluxes. The present review primarily focused on the physicochemical and a select few biological drivers that directly or indirectly govern these two fluxes. Our primary aim was to elucidate the spatiotemporal variability of the carbon biogeochemistry parameters and characterize the drivers of air–water CO_2_ and CH_4_ fluxes by reviewing the studies conducted in the Indian part of the Sundarbans (the estuaries adjoining the Indian Sundarbans).

## 2. Indian Sundarbans Mangrove Forest: A Brief Overview

Sundarbans is the world’s biggest continuous patch of mangrove forest, situated on the lower stretch of the Ganges–Brahmaputra–Meghna River delta system. It roughly covers an area of 10,200 km^2^, of which Bangladesh occupies 60% (Bangladesh Sundarbans Forest) and 40% remains in India (Sundarbans Biosphere Reserve) (UNESCO World Heritage Site: https://whc.unesco.org/en/list/798/, accessed on 2 October 2022). The Indian Sundarbans was declared a World Heritage Site and Biosphere Reserve by the International Union for Conservation of Nature (IUCN) and the United Nations Educational, Scientific, and Cultural Organization (UNESCO) in 1987 and 1989, respectively [56]. The Indian Sundarbans mangrove forest has one of the highest species diversity rates in the world, and it shelters several floras and faunas, including the iconic Royal Bengal Tiger. This mangrove forest provides many ecosystem services to the millions of people residing in its periphery. The reserve forest of the Indian Sundarbans covers an area of 4263 km^2^, including the core and buffer areas. The transitional zone covers an area of 5367 km^2^, densely populated with agricultural lands [57]. The soil texture in the Sundarbans varies from clayey to sandy loam to silty. The soil in the carbon-rich Sundarbans ecosystem accumulates 49–98% of the carbon storage within the depths of 0.5–3 m [26]. Several scholars have categorized the tropical climate in the Sundarbans as pre-monsoon (February to May), monsoon (June to September), and post-monsoon (October to January). The Sundarbans experience an average annual rainfall of ~1770 mm, 75% of which occurs during the monsoon. The ambient temperature shoots up to ~40 °C during the pre-monsoon season (in the summer months of May and June) and goes down to ~10 °C during the post-monsoon season (in the winter months of December and January). Samanta et al. [58] showed that between 2000 and 2020, the Indian Sundarbans lost 110 km^2^ of mangrove cover from the reserve forest area due to erosion. A genus composition study of the mangrove forest indicated a greater prevalence of salt-tolerant genera. The study showed an overall decline in forest cover and health conditions in the Indian Sundarbans. Increased salinity and temperature and reduced rainfall during pre-monsoon and post-monsoon periods were primarily responsible for such alterations in mangrove cover. Most rivers that fed this region with freshwater have become defunct, including Ichhamati, Jamuna, Bidyadhari, Noai, Suti, Kumarjol, Ghagramari, Karati, Thakuran, Bidya, Saptamukhi, and Matla [59,60]. The Hooghly River maintained the perennial freshwater flow into the Indian Sundarbans; however, the water flow is insufficient to rejuvenate the already decayed distributaries [59]. The Matla estuary is located in the central part of the Indian Sundarbans and flows through mangrove forests. The monsoon runoff and the tidal activity help to maintain its estuarine characteristics [61]. Both the Hooghly and Matla estuaries are meso-macro tidal, experiencing semidiurnal tides [62]. Besides the Hooghly and Matla Rivers, several other rivers flow through the Indian Sundarbans mangrove system, from west to east, such as the Mooriganga, Saptamukhi, Thakuran, and Bidya.

## 3. Overview of the Carbon Biogeochemistry Research in Indian Sundarbans

**Figure 1:**
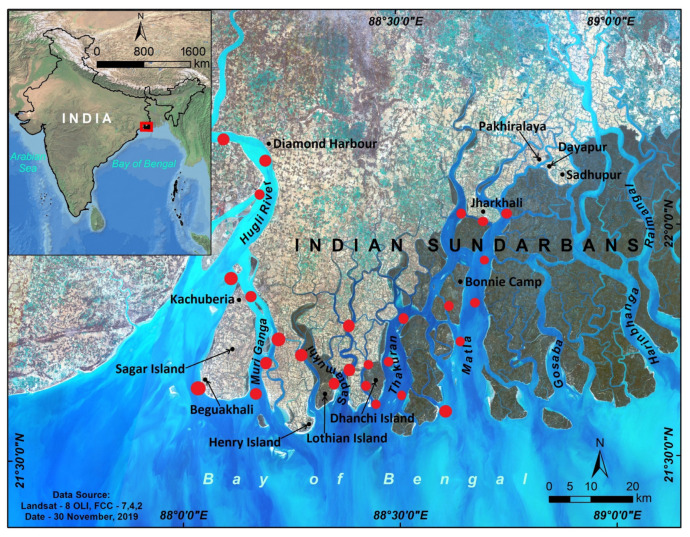
The Indian Sundarbans mangrove ecosystem and the adjacent estuaries. Black dots refer to the study locations, and the red dots denote the sites sampled in different studies and considered in this review article. The inset map shows the location of the Indian Sundarbans.

The Hooghly and Matla estuaries are the most studied regions in the Indian Sundarbans, a mangrove-dominated estuarine system (Figure 1). Mukhopadhyay et al. [63] studied the monthly variation of CO_2_ fluxes between the surface water of the Hooghly River and the associated atmosphere during 1999 (Table S1). Apart from CO_2_ fluxes, they also measured TAlk, salinity, dissolved oxygen (DO), gross primary production (GPP), and community respiration (CR). Later, Padhy et al. [64] developed an algorithm to compute the *p*CO_2_ and CO_2_ flux from remotely sensed data on sea surface temperature (SST) and chlorophyll *a* (Chl-*a*) concentration and in situ measurements in the Hooghly estuary. Several studies characterized the spatial and temporal (seasonal and diurnal) variations in CO_2_ fluxes in this region [65,66,67]. Akhand et al. [68] and Dutta et al. [69] compared the CO_2_ flux dynamics between the Hooghly and the Matla estuaries. The role of groundwater on inorganic carbon dynamics and *p*CO_2_ in the Hooghly and Sundarbans estuarine systems was revealed by Akhand et al. [70] using a coupled automated ^222^Rn and *p*CO_2(water)_ measurement system. All these studies also measured associated carbon parameters such as dissolved inorganic carbon (DIC) (Table S2), dissolved organic carbon (DOC), and particulate organic carbon (POC) (Table S3), along with pH, salinity, SST, TAlk, wind speed, and Chl-*a*. Besides the Hooghly and Matla estuaries, the Mooriganga, Saptamukhi, and Thakuran estuaries also received substantial attention regarding carbon biogeochemistry [71,72,73,74,75]. Several researchers also covered parts of Edward Creek, Herobhanga River, and Lothian Island [73,76] (Table S4). Relatively fewer studies are available on CH_4_ dynamics in the Indian Sundarbans (Table S5). Biswas et al. [77] studied the CH_4_ flux dynamics covering the Sundarbans estuaries (Muriganga, Saptamukhi, and Thakuran Rivers), Lothian Island, and the Hooghly estuary (Diamond Harbour, Kachuberia, Beguakhali) in 2003. This study reported the spatial and temporal changes in the CH_4_ fluxes. Neetha [78] compared the CH_4_ flux between Jharkhali Island (Sundarbans estuary) and the Hooghly estuary. Later, Dutta et al. [69,79,80,81,82] studied the CH_4_ flux around Lothian Island, the Hooghly estuary, and the Saptamukhi estuary.

## 4. Carbon Parameters and Total Alkalinity in Estuarine Water

Carbon parameters (DIC, DOC, and POC concentrations), TAlk, and other physicochemical parameters can regulate CO_2_ and CH_4_ fluxes. Researchers have extensively used the bulk formula method to estimate air–water CO_2_ and CH_4_ exchange rates in the Indian Sundarbans [83,84]. Computation of *p*CO_2(water)_ requires SST and sea surface salinity data, along with knowledge of DIC and TAlk (expressed in moles per kilogram of seawater), because the concentration of DIC and TAlk remains constant with changes in water temperature and pressure [85]. Different biogeochemical processes can influence the concentration of DIC in an aquatic system. Cycling of DIC is related to the production and utilization of CO_2_ through photosynthesis and respiration, i.e., the metabolic state of an estuary [86].

Apart from the heterogenic activities in a system, DIC can originate from riverine and groundwater inputs, inputs from inter-tidal wetlands, and photodegradation of dissolved organic matter [87,88,89,90]. Hence, to understand the net autotrophy (sink) or heterotrophy (source) nature of an estuary or an open water system, analysis of CO_2_ saturation is insufficient [91]. Thus, a detailed understanding of the sources, cycling patterns, and fate of DIC in the estuaries is needed to analyze the biogeochemical cycling of carbon and develop carbon budgets for coastal systems.

A significant fraction of the organic matter in estuarine and oceanic water remains dissolved [92,93,94]. Estuaries receive organic matter from adjacent land masses and local sources [95]. In the estuarine mixing processes, the terrestrial organic carbon undergoes rapid removal and decomposition [96]. Biodegradation is one of the primary processes responsible for removing DOC from estuarine and marine waters [97,98,99]. In many estuarine systems, respiration exceeds primary production [35,100], and the available organic matter is wholly or partially mineralized [98,99,101]. Thus, higher DOC may correlate with higher *p*CO_2_ concentrations [102].

Globally, most of the estuaries are net sources of CO_2_, and along with DIC and DOC, land-derived POC plays a vital role in determining that characteristic [103]. Annually, mangroves export 28 Tg C of POC to the sea. Plant debris, phytoplankton, and microphytobenthos generate this POC [104]. This POC in estuarine water causes heterotrophic activity and influences the DIC composition in seawater through mineralization [35,105].

The TAlk represents the inorganic carbon content in the aquatic body. It is the sum of bicarbonate, carbonate, borate, and hydroxide ions [83]. TAlk, when produced more than DIC, is often considered to increase the sinking potential for atmospheric CO_2_ since it increases the carbonate buffering capacity of seawater [106]. Rivers are the primary transporters of inorganic carbon [107]; moreover, they are a potent source of the same [48,108,109,110]. The coastal ocean’s source/sink characteristics depend on carbonate buffering capacity. Freshwater discharge, agricultural runoff, and groundwater discharge primarily regulate this buffering capacity [111,112]. Human activities such as deforestation, excessive agricultural practices, and events such as acid rain deposition cause a reduction in the carbonate buffering capacity of coastal systems [113,114]. Hence, along with other carbon parameters, the TAlk dynamics study is crucial in understanding coastal water’s CO_2_ source/sink behavior.

### 4.1. Spatial Variability

This region exhibited significant spatial variability in the carbon parameters and TAlk. Several studies have compared the DIC content of the anthropogenically disturbed Hooghly estuary with the less influenced Saptamukhi, Thakuran, and Matla estuaries (the Sundarbans estuary). They all reported higher DIC content in the Hooghly estuary than the others (Table S2). Ray et al. [84] observed similar findings from the Hooghly estuary and Lothian Island. Akhand et al. [70], while studying the influence of submarine groundwater discharge (SGD) on the DIC of estuarine water, reported about four times higher DIC content in the groundwater compared to the estuarine surface water. Though DIC has been well sampled throughout the spatial extent of this region, more endeavors are needed to measure the stable isotopic signatures of DIC to develop a better understanding of the DIC sources in this estuarine complex.

Compared to the DIC, fewer studies report DOC dynamics from the Indian Sundarbans region (Table S3). Ray et al. [73,84], Dutta et al. [67,69], and Akhand et al. [66,70] compared the DOC values for the Hooghly and Sundarbans estuaries. All of the studies documented similar values of DOC for the Hooghly and Sundarbans (Saptamukhi, Thakuran, and Matla) estuaries. According to the study by Ray et al. [73], rather than the DOC formation and decomposition rates, the lower residence time of water in the mangroves was responsible for the low value of DOC in the Hooghly and Sundarbans estuaries. Akhand et al. [70] reported the surface water and groundwater DOC in the Hooghly and Matla estuaries. In the Matla estuary, DOC in groundwater was significantly lower than the surface water. Thus, groundwater did not prove to be a significant source of DOC in the Sundarbans estuarine system. As mangrove habitats are considered the primary source of DOC in mangrove-adjacent estuaries, studies focusing on DOC variability from the mangrove island periphery to open estuarine channels are required to understand the rate of DOC dilution in these waters.

Ray et al. [73,84] compared POC between the Hooghly and Saptamukhi estuaries (Table S3). The studies suggested that POC in the Saptamukhi estuary has an autochthonous origin and that the Hooghly estuary is the primary source of terrestrial organic matter draining into Saptamukhi. The relatively higher POC in the Hooghly estuary compared to the Sundarbans estuaries (Saptamukhi, Matla) was attributed to increased freshwater flow in the estuary from upstream of the Hooghly River [66]. However, such measurements are required in all the major estuaries of this region to generate a more holistic scenario on this issue.

Several researchers studied the buffering capacity of the mangrove-dominated estuarine waters of the Indian Sundarbans (Table S2). Akhand et al. [76] studied the spatial variability of TAlk in the inner (Herobhanga), middle (Thakuran), and outer (Edward) estuaries. They did not observe variations in the TAlk values between the inner and middle estuaries; however, the outer estuary exhibited lower TAlk values. Akhand et al. [68] also reported similar results, where inner estuarine stations showed higher TAlk values than outer estuarine stations. Ray et al. [73] studied TAlk variability along the salinity gradient of the Hooghly estuary, the mangrove-dominated Saptamukhi estuary’s adjacent coastal waters of the Bay of Bengal, in 2014. However, they reported a narrow range of TAlk for both regions. Ghosh et al. [83] studied the Hooghly estuary (from the upper to the lower estuary) and the adjacent coastal waters during 2015–2016. They observed TAlk values decreasing from the upper to the lower estuary. Dutta et al. [67,69] studied TAlk variations in the Hooghly and Sundarbans estuaries and reported no spatial differences. However, the Hooghly estuary exhibited wider variations. Akhand et al. [70] compared the TAlk in the surface water and groundwater between the Hooghly and Matla estuaries. Both surface water and groundwater TAlk were marginally higher in the Hooghly estuary. Compared to surface water, TAlk was significantly higher in groundwater for both regions. Acharya et al. [71] studied TAlk in the Saptamukhi, Thakuran, and Matla estuaries, and they did not record any significant variation.

Several researchers have studied *p*CO_2_ in the Indian Sundarbans (Table S4). Sea surface temperature (SST) influences the *p*CO_2(water)_ by decreasing the solubility of CO_2_ in water [115]. The *p*CO_2(water)_ in the estuary decreased with proximity to the open sea [76]. *p*CO_2(water)_ in Edward creek (0 km from the shoreline), Thakuran River (31 km from the shoreline), and Herobhanga River (55 km from the shoreline) were 507.98 ± 73.69 µatm, 518.84 ± 71.94 µatm, and 234.34 ± 64.08 µatm, respectively; whereas, *p*CO_2(air)_ showed comparatively less variability (386.73 ± 1.97 µatm, 386.80 ± 5.05 µatm, and 376.26 ± 2.75 µatm, respectively). Ray et al. [84] studied the *p*CO_2_ in the riverine (992.5 µatm) and marine parts (372 µatm) of the Hooghly estuary and reported similar findings. Akhand et al. [68] compared the *p*CO_2(water)_ in the Hooghly and Matla estuaries during 2013–2014. Being an anthropogenically influenced estuary with a high river discharge, the annual mean *p*CO_2(water)_ value for the Hooghly estuary (~2200 µatm) was higher than the comparatively less anthropogenically disturbed Matla estuary (annual mean ~530 µatm). Dutta et al. [67] reported similar observations in the Hooghly (267 µatm to 4678 µatm) and Sundarbans estuaries (376 µatm to 561 µatm). *p*CO_2_ for the Hooghly estuary reduces gradually towards the offshore region. However, they did not report any spatial variation for the Sundarbans estuary [67]. Acharya et al. [71] studied the Saptamukhi, Thakuran, and Matla estuaries (Sundarbans estuary) from 2016 to 2020 and reported an average annual value of 993 ± 898 µatm. Though a substantial number of studies have been carried out in this regard, most endeavors indirectly estimated *p*CO_2(water)_, which adds to the uncertainties in flux computation. More endeavors are needed to measure *p*CO_2(water)_ directly and continuously through the deployment of automated sensors.

Several researchers characterized the spatial variation in CO_2_ flux between air and water (Table S4). Padhy et al. [64] measured the CO_2_ flux covering the entire Hooghly estuary. They observed significant variation in CO_2_ flux from the seaside to the upstream region due to the estuary’s north–south orientation. The estuary acted as a source with variable magnitude (90 mol C m^−2^ year^−1^ during summer and 0.5 mol C m^−2^ year^−1^ during winter). According to Akhand et al. [76], the inner (Edward Creek) and middle estuary (Thakuran River) acted as net sources (29.69 and 23.62 mg CO_2_ m^−2^ day^−1^ respectively), while the outer estuary acted as a net sink (−33.37 mg CO_2_ m^−2^ day^−1^). The heterotrophic nature of the water column and the sediments [116,117] added to community respiration in the mangrove estuary, which largely contributed to the CO_2_ source behavior of the inner and middle estuaries. At the same time, the dominant autotrophic characteristics of the outer estuary govern its sink behavior. Akhand et al. [68] compared the water-to-air CO_2_ emissions from the Hooghly and Matla estuaries. They reported that the annual mean CO_2_ emission from the Hooghly estuary was 14 times higher than that of the Matla estuary. The increased river runoff due to high river discharge was primarily responsible for the increased CO_2_ efflux from the Hooghly estuary during the last decade [68]. Dutta et al. [67,69,72] reported similar observations on CO_2_ efflux while working in the Hooghly and Sundarbans estuaries. Bacterial abundance in the highly nutrient-rich water of the urbanized river stretches triggered higher respiration and rapid mineralization [118], which in turn caused CO_2_ supersaturation and higher CO_2_ efflux [119,120]. Akhand et al. [65] compared the nature of CO_2_ flux between the creeks of Dhanchi Island and the adjacent estuary. According to the study, the creeks acted as net sources of CO_2_, while the estuary was a net sink for CO_2_. Similar to *p*CO_2(water)_, fluxes are estimated in most studies, which leaves a substantial scope of uncertainties. Direct flux measurements should be undertaken by future researchers by deploying chamber techniques to reduce the uncertainty in flux estimations.

Compared to other carbon parameters, fewer studies exist on the CH_4_ dynamics and air–water flux in Sundarbans estuarine water (Table S5). Biswas et al. [77] studied the spatial dynamics of CH_4_ in the Hooghly River estuary; in the stations, Diamond Harbour (inner estuary), Kachuberia (tip of Sagar Island, mid estuary), and Beguakhali (sea end/river mouth/outer estuary). Saptamukhi, Mooriganga, Thakuran, and the adjacent waters of the Lothian Island, were also studied. *p*CH_4_ in the Hooghly estuary was lower than in the Saptamukhi, Mooriganga, and Thakuran river systems, indicating the latter received a higher input of methane-rich waters from the surrounding mangroves. Dutta et al. [80] measured *p*CH_4_ in the surface water and porewater in the Saptamukhi estuary adjacent to Lothian Island. Compared to the groundwater, *p*CH_4_ was marginally higher in the surface water. Dissolved CH_4_ in water and CH_4_ flux were compared between the Hooghly and Sundarbans estuaries by Dutta et al. [69] during the pre-monsoon of 2016. The CH_4_ concentration in the Hooghly estuary varied over a broader range than in the Sundarbans estuary. In both estuaries, air–water CH_4_ flux was always positive, suggesting efflux from water to air. In the Hooghly estuary, maximum and minimum CH_4_ flux was reported from the freshwater and marine zones, respectively.

Table 1 summarizes the range of all the parameters reviewed in this study in the inner, middle, and outer estuaries of the Indian Sundarbans. TAlk and DIC exhibited almost similar spatial variability in these estuaries. The minimum observed concentrations of these two parameters were similar in the inner, middle, and outer estuarine reaches. However, the maximum values decreased from the inner to the outer estuarine regions. The decrease in DIC from the inner to outer regions was higher than the corresponding decrease in TAlk. This observation again indicates the influence of the coastal waters having a high TAlk/DIC ratio, implying a high buffering capacity. The DOC range in the inner estuarine region was significantly higher than the middle and outer reaches, primarily due to the allochthonous anthropogenic inputs from the upper reaches. The POC, on the contrary, showed higher ranges in the outer estuarine reaches. This observation indicates the presence of autochthonous POC derived from the mangrove environment and allochthonous POC outflown, primarily through the Hooghly estuary. *p*CO_2(water)_ and air–water CO_2_ flux mirrored each other in their spatial variabilities, which indicates that *p*CO_2(water)_ is the fundamental parameter that regulates the air-water CO_2_ fluxes in these estuaries. The maximum values of *p*CO_2(water)_ and air–water CO_2_ flux steadily decreased from the inner estuarine reaches to the outer region. This observation again unequivocally indicates that the seawater low in *p*CO_2(water)_ having a high buffering capacity leads to lower air-water CO_2_ fluxes in the outer regions compared to the inner reaches, where freshwater plays a dominant role. CH_4_ concentration in water and air–water CH_4_ flux showed a similar spatial trend. The inner estuarine region, having higher organic carbon than the middle and outer reaches, provided ample substrate for methanogenic activities, which led to higher dissolved CH_4_ levels in the water and eventually higher air–water CH_4_ fluxes.

### 4.2. Temporal Variability

This section discusses the diurnal and seasonal variabilities of the carbon biogeochemistry-related parameters reported by several studies. Padhy et al. [64] studied the DIC in the Hooghly estuary (Table S2) and the adjacent coastal waters following an empirical equation [121]. They reported lower values in May and November 2014 (990 ± 27 μmol kg^−1^ and 1130 ± 29 μmol kg^−1^, respectively) and relatively higher values in January, February, and September 2014 (1310 ± 107 μmol kg^−1^, 1711 ± 19 μmol kg^−1^, and 1697 ± 77 μmol kg^−1^, respectively). The relative riverine water fluxes, which bring terrestrial organic sources into the estuarine system, influenced the lower and higher DIC in the respective months. Dutta et al. [67,69] studied DIC variability in the Hooghly and Sundarbans estuaries during pre-monsoon and post-monsoon. A 6% increase in DIC was observed during pre-monsoon in the Hooghly estuary, while DIC did not vary much in the Sundarbans estuary seasonally. Ray et al. [84] also reported marginally higher values of DIC in the Hooghly estuary during pre-monsoon than post-monsoon. However, Akhand et al. [66] showed that pre-monsoon DIC concentrations were significantly higher than the monsoon and post-monsoon concentrations.

Dutta et al. [72] studied the diurnal dynamics of DOC in the Sundarbans estuary. DOC showed non-conservative behavior during low tide and conservative behavior during high tide (Table S3). The moderately significant relation between DOC and salinity indicated that the DOC in the Sundarbans estuarine system remains regulated by processes other than estuarine mixing. The biogeochemical and physiological processes, along with the photo-oxidation in the mangrove system, were suggested to regulate the diurnal fluctuations in DOC. Dutta et al. [67,69] reported the seasonal changes in DOC during the pre-monsoon and post-monsoon seasons. In the Hooghly and Sundarbans estuaries, the pre-monsoon DOC concentrations were 48% and 45% higher than the post-monsoon DOC concentrations. However, Akhand et al. [66] did not observe any such trend in the DOC.

Dutta et al. [69,72] reported the seasonal and diel changes in POC, respectively (Table S3). In the Hooghly estuary, POC was higher by 52% during pre-monsoon than post-monsoon, while in the Sundarbans estuary, POC was higher during post-monsoon (by 62%) when compared to pre-monsoon. The diurnal study showed marginally higher POC in the Sundarbans estuary during the daytime. A seasonal study by Ray et al. [84] also reported higher POC in the waters surrounding Lothian Island during post-monsoon (45.4 ± 7.5 µM) than pre-monsoon (28.0 ± 8.6 µM). The diurnal variation study of POC [84] showed an increasing POC trend with increasing water height. Like DIC, Akhand et al. [66] reported significantly higher POC levels in the Matla estuary during the pre-monsoon season compared to the other two seasons.

The temporal variability of TAlk is a relatively less documented topic in the carbonate chemistry research in the Indian Sundarbans (Table S2). Akhand et al. [68] studied the seasonal variation of TAlk in the Hooghly and Matla estuaries, which showed lower values of TAlk during the monsoon in the inner estuarine stations from both study areas. Ghosh et al. [83] reported similar observations from the Hooghly estuary (from the upper to the lower estuary) and the adjacent coastal waters. The TAlk values were highest during the pre-monsoon and lowest during the monsoon. Dutta et al. [72] studied the diurnal variability of TAlk in the Sundarbans estuary (Saptamukhi, Thakuran, and Matla estuaries), which varied over a narrow range (2.19 to 2.58 m equiv/l). Akhand et al. [66] also reported similar observations from the Matla estuary (2178 to 2273 μmol kg^−1^). They sampled twice during the two consecutive pre-monsoon seasons of 2017 and 2018 and observed significantly higher mean TAlk than in the monsoon and post-monsoon seasons.

Biswas et al. [74] measured the diurnal variation of *p*CO_2_ in the Mooriganga, Saptamukhi, and Thakuran estuaries in 2001 (Table S4). *p*CO_2(air)_ varied with the time of day (minimum at 1200 h and maximum at 2100 h) and ranged between 403 µatm and 635 µatm, respectively. The *p*CO_2(water)_ was highest at 0600 h (1062 µatm) and lowest at 1500 h (416 µatm). While mangrove plant cover, wind velocity, and turbulence influenced the *p*CO_2(air)_, tide-driven changes in salinity regulated the *p*CO_2(water)_ variation. Padhy et al. [64] studied *p*CO_2_ in the Hooghly estuary and the adjacent coastal waters during the winter and summer of 2008. During winter, *p*CO_2(water)_ ranged between 320 µatm and 500 µatm in the coastal domain and 340 µatm to 375 µatm in the upstream river. However, during summer, *p*CO_2(water)_ was spatially uniform (about 450 µatm). Changes in tidal circulations and physicochemical properties along the estuary (from the upstream river to the offshore estuary) caused spatial variation in *p*CO_2(water)_. Ray et al. [84] studied *p*CO_2_ in the adjacent waters of Lothian Island and throughout the Hooghly River (from the upstream riverine part to the river mouth) during the pre-monsoon and post-monsoon of 2014. The study reported comparatively higher values of *p*CO_2_ during the pre-monsoon season. Akhand et al. [66] reported the highest mean *p*CO_2(water)_ during the monsoon and the lowest during the post-monsoon season.

Mukhopadhyay et al. [63] studied the air–water CO_2_ flux along the Hooghly estuary in 1999. The study indicated the estuary as a source of CO_2_ during the pre-monsoon (the maximum emission rate being 84.4 mmol m^−2^ d^−1^), and the role reversed during the monsoon (the maximum influx rate being −2.78 mmol m^−2^ d^−1^). (Table S4). Biswas et al. [74] studied the diurnal variation of air–water CO_2_ flux in the Sundarbans estuary (Mooriganga, Saptamukhi, and Thakuran estuaries) in 2001. The study showed a distinct diurnal variation in the CO_2_ flux (from −16.2 to 49.9 µmol m^−2^ h^−1^). Ambient and sea surface temperatures, daylight hours, and the rate of photosynthetic activity influenced the rate of CO_2_ flux. Akhand et al. [76] studied the diurnal variation in CO_2_ flux in the Edward Creek, Thakuran River, and Herobhanga River estuaries for their distance from the coastal waters. The study provided insight into the changing behavior of the estuarine system in terms of CO_2_ flux from the inner to outer estuarine regions. The findings about the diurnal variability were similar to those observed by Biswas et al. [74]; however, apart from the photosynthetic activity and respiration, the semidiurnal tidal cycles were responsible for the variations. Chen et al. [122] also observed that the strong CO_2_ source nature in the upper estuaries often turns into a CO_2_ sink character in the outer estuarine river plumes. In line with the pCO_2(water)_-related observations made by Akhand et al. [66], they reported air–water CO_2_ fluxes decreasing from monsoon to pre-monsoon, followed by post-monsoon seasons.

Biswas et al. [77] measured the seasonal changes in CH_4_ concentration in water and air–water CH_4_ flux in the Hooghly estuary (from the inner riverine zone to the river mouth), the Saptamukhi, Mooriganga, and Thakuran estuaries, and the adjacent waters of Lothian Island. Seasonal variability of CH_4(air)_ was prominent; however, it spanned over a narrow range (Table S5). The CH_4(water)_ was maximum during the post-monsoon and minimum during the monsoon season. Increased dissolved CH_4_ concentration was during high tide and vice versa. As observed by other researchers, the CH_4_ concentration in estuarine rivers generally shows a decreasing trend from freshwater to saltwater regions [123,124,125]. However, in the Hooghly estuary, the CH_4_ concentration was observed to increase in the lower stretch of the estuary. Apart from the riverine source, the mangrove ecosystem in the lower stretch of the estuary acted as an additional source of CH_4_; hence, the CH_4_ concentration increased. The primary peak in CH_4_ flux was during the post-monsoon, and a secondary peak was during the monsoon. The residence time of CH_4_-rich riverine water in the estuary plays an essential role in CH_4_ flux. Dutta et al. [81,82,85,86] recorded similar results from the Saptamukhi estuary. CH_4_ concentration was maximum during the post-monsoon and minimum during the pre-monsoon. CH_4_ flux was water-to-air throughout the year, and maximum efflux occurred during the monsoon and minimum during the pre-monsoon. Padhy et al. [126] studied the CH_4_ emissions from the surrounding waters of degraded mangroves. Based on remote sensing analysis, they considered Sadhupur, Pakhiralaya, and Dayapur as degraded mangrove sites for their study. They measured air–water CH_4_ fluxes in stagnant water, during tidewater, and after tidewater. In all the locations, the CH_4_ concentration and the CH_4_ flux were higher in stagnant water during the monsoon.

Though an adequate number of studies characterized the seasonal variability of the carbon biogeochemistry parameters discussed above, long-term temporal monitoring over the course of decades at a fixed location is absent from this region. Systematic monitoring of these parameters in high and low-tide sessions during spring tide and neap tide phases over multiple years would enable us to characterize the changes in the concentrations of these parameters over time. Such prolonged monitoring would allow us to correlate the changes in concentrations of these parameters with climate change indicators and/or anthropogenic activities. In the absence of such long-term monitoring, the role of climate change and/or anthropogenic activities in governing the carbon biogeochemistry in these regions remains unanswered.

Table 2 summarizes the range of all the parameters reviewed in this study in the pre-monsoon, monsoon, and post-monsoon seasons. The maximum concentrations of TAlk and DIC occurred during the pre-monsoon season, followed by the post-monsoon and the monsoon seasons. This observation indicates that these two parameters remained high during the dry seasons (pre-monsoon and post-monsoon) and underwent dilution in the monsoon season. DOC and POC also showed similar trends, which indicates that during the dry season, when evaporation rates remain high with minimal freshwater discharge from the upper reaches, the estuarine water mass remains enriched with both of these forms of organic carbon. However, during the monsoon, these values remain lower primarily due to the dilution effect in the presence of voluminous freshwater flow. *p*CO_2(water)_, on the contrary, exhibited higher concentration ranges during the monsoon season. The dominance of riverine freshwater and catchment flow triggered by the monsoon-induced rainfall reduces the pH of the estuarine water column, which facilitates a higher *p*CO_2(water)_ than that observed in the dry seasons. Air–water CO_2_ fluxes reciprocated accordingly, and the highest flux ranges occurred during the monsoon, followed by the pre-monsoon and post-monsoon seasons. The pre-monsoon season, which coincides with the summer months, experiences enhanced temperatures that reduce the solubility of CO_2_ in the estuarine water and give rise to substantially higher magnitudes of air–water CO_2_ flux. However, the effect of enhanced temperature (during the pre-monsoon season) could not overrule the effect of lowered pH levels (during the monsoon season) in regulating *p*CO_2(water)_. In the case of CH_4_ concentrations, however, the pre-monsoon season exhibited higher ranges, as did the air–water CH_4_ fluxes. This observation showed that higher temperatures activated methanogenic activities more in the pre-monsoon season than in the other two seasons.

## 5. Factors Regulating Air-Water CO_2_ and CH_4_ Flux

Various factors governed the air–water CO_2_ and CH_4_ flux in the Indian Sundarbans mangrove system. For ease of discussion, we categorized them into three major types: physical, biogeochemical, and hydrological.

### 5.1. Role of Physical Factors

Water temperature, wind speed, tidal activity, rainfall, and photosynthetically active radiation (PAR) are critical in regulating CO_2_ and CH_4_ concentrations in water and, subsequently, the CO_2_ and CH_4_ fluxes. Water temperature is known to influence CO_2_ solubility in seawater [127] and, subsequently, the variations of *p*CO_2(water)_ [128]. Almost all the aforementioned studies that monitored the seasonal variability of *p*CO_2(water)_ and air–water CO_2_ fluxes observed higher magnitudes of both these parameters during the pre-monsoon season compared to the post-monsoon season [66,68]. However, attributing only temperature as the sole factor in regulating *p*CO_2(water)_ would be erroneous, as several other interlinked physical factors play crucial roles.

The availability of PAR regulates autotrophic activities in estuarine water [129]. Reduced PAR causes dominance of heterotrophy and induces CO_2_ efflux [130]. Increased turbidity or total suspended matter in the seawater reduces PAR penetration in the water column [131], increasing the CO_2_ source potential of seawater. Akhand et al. [68] measured PAR in the Hooghly and Matla estuaries. They reported a significant difference in PAR between the two estuaries, with substantially lower PAR in the Hooghly compared to the Matla estuary. This low PAR was mainly due to the high turbidity levels in Hooghly compared to Matla. Sadhuram et al. [132] reported that the estuarine water column of the Hooghly remains highly turbid almost throughout the year due to strong tidal and wave actions, which leads to the bottom-churning of sediments. Tidal cycles also play a crucial role in regulating *p*CO_2(water)_ as they cause the physical mixing of the seawater with the riverine freshwater. Akhand et al. [65,68] observed that freshwater from upstream had a higher *p*CO_2(water)_, whereas the seawater encroaching during the high tide had a low *p*CO_2(water)_. Thus, it is an essential driving factor for CO_2_ flux. During high tide phases, due to the dominance of seawater in the estuary, CO_2_ efflux remains low.

The CH_4_ concentration in estuarine water also varies with tidal cycles. During high tides, flushing the mangrove swamp brings large quantities of CH_4_ into the estuary, causing an increased CH_4_ concentration in the water [77]. Wind speed is another physical forcing factor that regulates flux magnitude. Higher wind speed facilitates CO_2_/CH_4_ exchange at the air–water interface, increasing flux magnitude [76]. Rainfall also plays a vital role in regulating flux in the estuarine region. Increased rainfall leads to increased river runoff and more drainage of mangrove porewater into the estuary. Thus, higher CO_2_ efflux generally prevailed during the monsoon seasons [66,68]. During the dry seasons, high levels of air pollution from the urban sectors are advected into the coastal regions, causing a gradual decrease in CH_4_ concentration in the air due to atmospheric oxidation [77].

### 5.2. Role of Biogeochemical Factors

Salinity is one of the significant factors that govern CO_2_ flux. Higher salinity means the dominance of seawater, where *p*CO_2_ is low [133]. Thus, CO_2_ efflux from the seawater also remains low. Decreased salinity in a coastal region refers to an increased river runoff or pore water discharge, rich in *p*CO_2_, promoting CO_2_ efflux [134]. The DIC in water mainly remains in carbonate and bicarbonate forms. Lower pH induces excess DIC to transform into a gaseous form, which eventually releases into the surrounding atmosphere through efflux. Akhand et al. [66] and Dutta et al. [72] observed a significant negative correlation between *p*CO_2(water)_ and pH.

The carbonate buffering capacity of estuaries depends on the TAlk/DIC ratio [135]. The higher this ratio, the lower the potential of CO_2_ emissions from the estuaries. Akhand et al. [65] observed that the northern Bay of Bengal waters has high TAlk/DIC, which enhances the carbonate buffering capacity within the estuaries during the high tide. This phenomenon prevents the conversion of carbonate and bicarbonate in water into excess DIC, reducing CO_2_ efflux from water to air. Chen et al. [136] also reported that mixing with seawater is the primary cause for the *p*CO_2_ to decrease near the river mouths. Akhand et al. [65] also noted that DOC and POC degradation to DIC occurs in the Sundarbans estuaries primarily through denitrification. The mangrove pedosphere offers a platform where organic matter degradation eventually enhances the DIC levels in the adjacent estuary, reducing carbonate buffering capacity [116].

Salinity shows a strong negative correlation with CH_4_ concentration in water, indicating riverine freshwater is the primary source of CH_4_ [137]. In several instances, higher chlorophyll concentrations also lead to higher levels of dissolved CH_4_ in water. Higher chlorophyll ensures increased productivity (provided all the other parameters such as water surface temperature, PAR, salinity, and pH are optimum), which eventually increases the organic matter supply to the sediments, and this causes increased methane production by methanogens [138].

### 5.3. Role of Hydrological Factors (Pore Water, Groundwater, and Freshwater Discharge)

Compared to the surface water, *p*CO_2_ and *p*CH_4_ in the pore water remain several folds higher [119]. Pore water and groundwater are often responsible for the high *p*CO_2_ and *p*CH_4_ in mangrove estuaries during low tide [139]. Microbial and root respiration in the rhizosphere and anaerobic methane emission driven by methanogens enhance the CO_2_ and CH_4_ levels in the porewater [9,79]. Das et al. [140] reported that porewater from the mangrove sediments of the Sundarbans has substantial potential to enhance the *p*CO_2_ and *p*CH_4_ in the adjacent estuaries. Studies indicate that pore water outflux from the sediments caused *p*CO_2_ to be high in the creeks during low tide [65]. However, this effect of pore water is not visible in the estuaries, a) due to the higher volume of water in the estuaries, which dilutes the pore water, and b) because the higher residence time of water in the creeks causes organic matter degradation, which further increases *p*CO_2_. Higher CH_4_ concentration in the estuarine water during post-monsoon is caused by the flux of pore water from the mangrove swamp sediments after monsoon rainfall and higher litter fall during post-monsoon [77]. This pore water laden with CH_4_ increases the CH_4_ concentration in estuarine water, causing maximum CH_4_ flux in the post-monsoon season.

Groundwater discharge is another process that influences the *p*CO_2_ in estuarine and oceanic waters [141]. Groundwaters are generally rich in CO_2_ [142]. Thus, it can significantly increase the *p*CO_2(water)_ and CO_2_ efflux in the discharged area. Groundwaters are also laden with nutrients and organic matter, which further increase the organic load in the water, increasing *p*CO_2_. However, Akhand et al. [70] did not observe any significant difference in DOC between groundwater and surface water in the Hooghly and Sundarbans estuaries. Groundwaters are also high in TAlk and DIC content, adding to CO_2_ flux. Akhand et al. [70] found TAlk and DIC in recirculated and fresh groundwaters four times higher than the surface waters in the Hooghly and Matla estuaries. They also observed that low-tide sessions had higher Radon-222 signatures, indicating higher groundwater contribution, which coincided with higher *p*CO_2(water)_. Akhand et al. [70] could not quantify the submarine groundwater discharge in the Sundarbans estuaries and, hence, could not assess the exact contribution of groundwater in enhancing estuarine *p*CO_2(water)_. However, their findings strongly indicate that groundwater seepage in these estuaries could play a pivotal role in regulating its *p*CO_2(water)_ and air–water CO_2_ fluxes, as groundwater samples had a significantly higher TAlk/DIC ratio than surface waters, which can significantly increase the estuarine water’s buffering capacity as well. Freshwater discharge from the upper reaches is crucial in regulating the *p*CO_2_ dynamics in estuarine regions worldwide [143]. In the Indian part of the Sundarbans (where carbon-biogeochemistry-related studies mostly concentrate), freshwater mainly comes from the Hooghly estuary on the western margin and the Raimangal in the east. The estuaries that flow through the central part of this mangrove forest receive minimal freshwater and retain their estuarine character mainly through monsoon-induced rainfall and runoff from upper catchment areas [61]. Akhand et al. [68] explicitly portrayed that higher riverine discharge into the Hooghly estuary led to four times higher *p*CO_2(water)_ and fourteen times higher CO_2_ emission in the Hooghly compared to the Matla estuary, which does not receive much freshwater and flows through the central part of the Sundarbans. Akhand et al. [68] also correlated the observed monthly mean *p*CO_2(water)_ and air–water CO_2_ flux in the Hooghly estuary with the monthly mean freshwater discharge reported by Rudra [144]. They observed that both mean *p*CO_2(water)_ and air–water CO_2_ flux increased with freshwater discharge. The highest CO_2_ effluxes were during the monsoon season, when the discharge peaked, and vice versa. Akhand et al. [68] concluded an undeniable role of riverine allochthonous DIC and organic matter input that enhanced *p*CO_2(water)_ with increasing riverine freshwater discharge. The same holds for Sundarbans estuaries, as well; however, riverine freshwater input data for the Sundarbans estuaries are unavailable, which barred several researchers from quantifying the role of riverine freshwater in governing the carbon dynamics in these estuaries.

The existing set of studies was, to a large extent, successful in characterizing the role of physical, biogeochemical, and hydrological factors in regulating CO_2_ and CH_4_ fluxes; however, future research should strive to acquire more data, as sampling number and frequency are the two major constraints at present. Given the vast expanse of this estuarine complex, the sampling stations should be randomly distributed throughout the estuarine system, and future research should focus on high-temporal-resolution sampling from each station to increase the number of observations that can reduce the uncertainties in the observed relationships.

### 5.4. Role of the Microbiomes in Carbon Dynamics of Sundarban

Microbiomes play a crucial role in carbon dynamics and biogeochemistry through the degradation of organic matter and through mineralization pathways. The CO_2_ and CH_4_ evasion caused by the microbiome-dependent processes mainly facilitates offsetting the carbon sequestration ability in the mangrove ecosystem, especially in the adjacent soil and water. In the Sundarbans, the microbiomes concerning carbon dynamics and sequestration have been relatively poorly studied compared to other counterparts of the mangrove ecosystem. Ghosh and Bhadury [145] studied coastal bacterioplankton diversity concerning biogeochemical cycling on the largest island of the Indian Sundarban delta, Sagar Island, using a 16S rRNA clone library and Illumina MiSeq techniques. They found several sequences belonging to Sphingomonadales, Chromatiales, Alteromonadales, Oceanospirillales, and Bacteroidetes, which might have a regulatory role in coastal carbon cycling. Among the bacterioplankton, an abundance of *Synechococcus* sp. during the monsoon period signified the role of oxygenic photoautotrophs in the coastal carbon cycling of the Sundarbans. Bhadury and Singh [146] further confirmed the dominance of *Synechococcus*-like 16S rRNA sequences and the influence of small-sized picocyanobacterial cells in regulating carbon export in the Sundarbans’ mangrove ecosystems while working in the Mooriganga estuary.

They also reported other cyanobacterial sequences showing taxonomic affiliation with members of the Chroococcales, Pleurocapsales, Oscillatoriales, and Stigonematales. Das et al. [147,148] reported that organic carbon was the most significant factor that regulated the total microbial population in the mangrove-adjacent sediment. They also depicted that cellulose-degrading bacteria dominated throughout the year in the sediment, which might have a decisive role in the mineralization and decomposition processes, majorly affecting the carbon biogeochemistry of the Sundarbans. Mukherjee et al. [149] conducted a post-monsoonal study in the Saptamukhi and Thakuran estuaries of the Sundarbans on bacterioplankton concerning inorganic nutrients and carbonate variables. They stated that Proteobacteria dominated the bacterioplankton, with a contribution from Bacteroidetes in the Saptamukhi and Cyanobacteria and Actinobacteria in the Thakuran; the interactions between physicochemical parameters, nutrient levels, and estuarine carbonate chemistry controlled post-monsoonal bacterioplankton abundance. Dhal et al. [150] revealed microbial communities in the waters of the “Island of Sundarban Mangroves” and “Open Marine Water” of the Thakuran-Matla River estuarine complex adjacent to the Maipith coastal area. They reported dominance of marine hydrocarbon-degrading bacteria under families Oceanospirillaceae and Spongiibacteraceae, where the most abundant bacterial family Rhodobacteracea almost equally dominated in both study sites. Their investigation further reported Actinobacteria, which is known to be associated with carbon cycling to decompose the plant biomass via degrading the cellulose and hemicellulose materials (a dominant resource material in mangrove plants).

## 6. The Lateral Flux of Carbon to the Bay of Bengal

The last two decades witnessed several studies that characterized the lateral fluxes of carbon from the terrestrial sectors to the adjacent ocean through the estuaries [8,151,152]. Recent developments in understanding estuarine carbon biogeochemistry indicate that long-term carbon sinks should encompass the laterally exported DIC from the estuaries to the nearshore coastal oceans [153]. The lateral export of organic matter and nutrients from the mangrove sediments to the estuarine waters plays a vital role in regulating the carbon flux. In this regard, Mukhopadhyay et al. [87] were the first to quantify the lateral fluxes of nutrients and DIC from the Hooghly estuary to the adjacent northern Bay of Bengal by deploying a biogeochemical mass balance model. They reported a lateral flux of DIC of 2.3 × 10^11^ mol per year while working from 1999 to 2001. Mukhopadhyay et al. [87] observed that 7.5% of the DIC was removed from the system as air–water CO_2_ flux during this lateral transport. Ghosh et al. [83], during 2015–16, observed that annually, the Hooghly estuary outfluxes around 4.45 ± 1.90 × 10^11^ mol and 4.59 ± 1.70 × 10^11^ mol of TAlk and DIC, respectively, to the adjacent coastal ocean. They concluded that the enhanced discharge load of inorganic and organic matter in the upper reaches of the Hooghly River led to an increased lateral flux of DIC and DOC, compared to the observations of Mukhopadhyay et al. [87]. Ghosh et al. [83] also inferred that the lateral DIC fluxes to the coastal ocean were 30 to 60 times higher than the air–water CO_2_ fluxes from the Hooghly estuary towards the atmosphere. Ray et al. [84] quantified the lateral fluxes of DIC, DOC, and POC from the Hooghly estuary and the Sundarbans estuaries as baseline data. They reported a lateral DIC, DOC, and POC flux of 3.45 × 10^11^ mol, 0.28 × 10^11^ mol, and 0.05 × 10^11^ mol per year, respectively, from the Hooghly estuary. In contrast, the Sundarbans estuaries outwelled 3.07 × 10^11^ mol, 2.52 × 10^11^ mol, and 0.48 × 10^11^ of DIC, DOC, and POC mol per year, respectively, with a total of 7.3 Tg C yr^−1^. Thus, Ray et al. [84] concluded that the mangrove-adjacent waters of the Sundarbans estuaries outwelled slightly less DIC but nine times more DOC and 9.6 times more POC than the Hooghly estuary. One of the constraints of this study [84] was the use of river discharge data from the Hooghly estuary to compute the lateral fluxes from the Sundarbans estuaries, which might lead to substantial uncertainties. Thus, further studies are of the utmost necessity to characterize the overall carbon budget of the Sundarbans estuaries.

## 7. Summary and Conclusions

**Figure 2:**
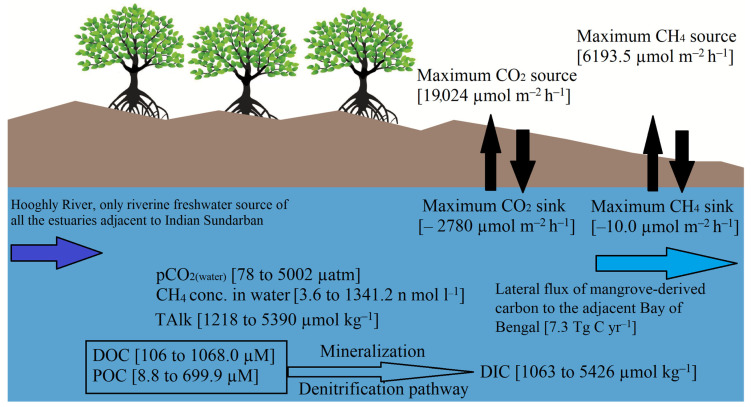
Schematic diagram of the ranges of different carbon parameters [particulate organic carbon (POC), dissolved organic carbon (DOC), and dissolved inorganic carbon (DIC)], total alkalinity (TAlk), partial pressure of CO_2_ (*p*CO_2_), and concentration of CH_4_ in the Indian Sundarbans’ adjoining estuaries according to different studies. Hooghly River is the only source of riverine freshwater in the Indian Sundarbans. The major organic matter degradation/ mineralization pathway is denitrification. The mangrove surrounding waters of these estuaries acted as a source and sink for CO_2_ and CH_4_ depending on the seasonality and spatiality; the source and sink capacity varied over a wide range. These estuaries also transfer a substantial amount of carbon to the adjacent Bay of Bengal, mainly in POC, DOC, and DIC forms.

Collating all the observations considered in this review, we can conclude that the *p*CO_2(water)_ and *p*CH_4(water)_ in the Sundarbans estuaries remain supersaturated compared to the atmospheric concentrations. Hence, these estuaries act as sources of CO_2_ and CH_4_. Figure 2 summarizes the entire review in a nutshell. The inner estuarine regions emit more CO_2_ and CH_4_ than the outer reaches close to the northern Bay of Bengal. Some studies indicated that the outer reaches could sometimes act as a sink for CO_2_, which is unusual for mangrove-adjacent waters. Analyzing the reasons behind this observation, several authors noted that the water from the adjacent Bay of Bengal is primarily responsible for enhancing the carbonate buffering capacity of these estuaries, especially in regions with an adequate quantity of riverine freshwater that does not end up in the upper reaches. Among the physical drivers, water temperature and photosynthetically active radiation play a pivotal role in governing CO_2_ emissions. Some significant biogeochemical and hydrological factors that governed the CO_2_ fluxes were salinity, porewater, groundwater, and freshwater discharge. The degree of primary productivity was the main regulating factor for air–water CH_4_ fluxes.

This review indicates that a substantial number of studies characterizing the air–water CO_2_ and CH_4_ fluxes exist in the estuaries of the Sundarbans. However, all these studies concentrated only on the estuaries that flow through the Indian counterpart of the Sundarbans. An adequate number of studies in the Bangladesh Sundarbans would enable us to draw a scenario about the entire Sundarbans estuarine network. Each major estuary flowing through the Sundarbans has unique freshwater–seawater admixing dynamics and a varying influence of adjacent mangroves. The present observations indicate that such subtle differences in the salinity regime and mangrove influence might lead to varying partial pressures of CO_2_ and CH_4_ [*p*CO_2(water)_ and *p*CH_4(water)_] and air–water CO_2_ and CH_4_ fluxes. Thus, sampling all the major waterways in the vast Sundarbans is necessary. Among the Indian estuaries, the Hooghly (flowing through the western boundary and carrying the bulk load of freshwater) and the Matla (flowing through the central part with minimal freshwater from the upper reaches) have received the most scientific attention. Other estuaries such as Raimangal, Bidya, Saptamukhi, and Thakuran should receive similar attention in the future.

The studies conducted so far have had varying sampling strategies. Some studies concentrated on fewer sampling points but stressed high temporal resolution, whereas others tried to cover more locations with a lower temporal resolution. In a dynamic estuarine environment such as the Sundarbans, tide-induced variability in carbon-biogeochemistry-related parameters poses a challenge to characterizing the mean and range of a particular parameter. Thus, more studies focusing simultaneously on the spatial extent and temporal resolution can alleviate the uncertainties in the data range. Besides the estuaries of the Bangladesh Sundarbans, which have yet to receive proper scientific attention in this regard, the estuarine waters flowing through the core area of the Indian Sundarbans also need rigorous sampling in the future, or else a substantial part of this unique eco-region remains undersampled. Whatever data on freshwater discharge are available at present mainly focus on the Hooghly estuary. Future researchers should model the estuarine flow through the various other estuaries of the Sundarbans that do not have a proper connection with the upper reaches. This would help in delineating the lateral fluxes of various forms of carbon from this system to the adjacent ocean and vice versa. Most of the studies accomplished so far have relied on discrete sampling techniques, which constrained the number of observations, as they require manual presence. Automated sensors on fixed platforms and the deployment of buoys should be on future research agendas. Otherwise, characterizing the temporal changes in the behavior of these estuaries with the changing climate and anthropogenic influences upstream would be highly challenging.

## Figures and Tables

**Table 1:** The range (minimum to maximum) of TAlk, DIC, DOC, POC, *p*CO_2(water)_, air–water CO_2_ flux, CH_4_ concentration in water, and air–water CH_4_ flux observed in the inner, middle, and outer estuarine reaches of the Indian Sundarbans.

Parameters	Inner Estuary	Middle Estuary	Outer Estuary
TAlk (µmol kg^−1^)	1219–5391	1369–4549	1218–3189
DIC (µmol kg^−1^)	1063–5126	1240–4111	990–2882
DOC (µmol L^−1^)	226.9–1068	249–263.6	10.6–328.2
POC (µmol L^−1^)	8.8–62.3	43.2–129.7	27–699.9
*p*CO_2(water)_ (µatm)	204–5851	279–2786	98–2015
Air–water CO_2_ flux (µmol m^−2^ h^−1^)	−301–16944	−249–9743	−860–7230
CH_4_ conc. in water (nmol L^−1^)	19.67–445.7	17.25–53.5	3.6–90.91
Air–water CH_4_ flux (µmol m^−2^ h^−1^)	−10.0–2801.2	61.6–3658.4	0.3–6193.5

**Table 2:** The range (minimum to maximum) of TAlk, DIC, DOC, POC, *p*CO_2_(water), air–water CO_2_ flux, CH_4_ concentration in water, and air–water CH_4_ flux observed in the pre-monsoon, monsoon, and post-monsoon seasons in the Indian Sundarbans.

Parameters	Pre-Monsoon	Monsoon	Post-Monsoon
TAlk (µmol kg^−1^)	1356–5391	1473–3300	1218–4419
DIC (µmol kg^−1^)	990–5126	1084–3431	1063–4469
DOC (µmol L^−1^)	82.7–1068	91.8–249.2	10.6–662
POC (µmol L^−1^)	11–699.9	43–87.3	8.8–457
*p*CO_2(water)_ (µatm)	143–3295	123–5851	98–4803
Air–water CO_2_ flux (µmol m^−2^ h^−1^)	−860–16,944	−350–19,024	−290–9448
CH_4_ conc. in water (nmol L^−1^)	15.4–445.7	13.65–327.2	3.6–117.2
Air–water CH_4_ flux (µmol m^−2^ h^−1^)	0.3–6193.5	14.2–515.06	−10.0–1230.5

## Data Availability

The data presented in this study are available in the supplementary material.

## Supplementary Materials

The following supporting information can be downloaded at: https://www.mdpi.com/article/10.3390/life13040863/s1, Table S1. The list of studies on air-water CO_2_ and CH_4_ fluxes, along with other related carbon-biogeochemistry parameters carried out in the Indian Sundarbans estuaries. Table S2. List of observations on TAlk and DIC from several studies on the Indian Sundarbans estuaries. The results are displayed either as mean ± standard deviation from the mean or as the range (minimum to maximum). NA denotes not available. Table S3. List of observations on DOC and POC from several studies on the Indian Sundarbans estuaries. The results are displayed either as mean ± standard deviation from the mean or as the range (minimum to maximum). Single magnitudes, in some instances, represent the mean (without any reported standard deviation). Table S4. List of observations on *p*CO_2(water)_ and air-water CO_2_ fluxes from several studies on the Indian Sundarbans estuaries. The results are displayed either as mean ± standard deviation from the mean or as the range (minimum to maximum). Single magnitudes, in some instances, represent the mean (without any reported standard deviation). Table S5. List of observations on CH_4_ concentration in water and air-water CH_4_ fluxes from several studies on the Indian Sundarbans estuaries. The results are displayed either as mean ± standard deviation from the mean or as the range (minimum to maximum). Single magnitudes, in some instances, represent the mean (without any reported standard deviation).
