# Supplementary material for: Carbon Biogeochemistry of the Estuaries Adjoining the Indian Sundarbans Mangrove Ecosystem: A Review
Source: Life (Basel). 2023 Mar 23;13(4):863. doi: 10.3390/life13040863 (PMC10141991; doi:10.3390/life13040863)
Supplement: Supplementary file 1 [file life-13-00863-s001.zip › life-2209708-supplementary.pdf]

**Supplementary Files**

*Review*

# Carbon Biogeochemistry of the Estuaries Adjoining the Indian Sundarbans Mangrove Ecosystem: A Review

**Isha Das [1,*], Abhra Chanda [1], Anirban Akhand [2] and Sugata Hazra [1]**

1 School of Oceanographic Studies, Jadavpur University, Kolkata 700032, India
2 Department of Ocean Science, Hong Kong University of Science and Technology, Kowloon, Hong Kong SAR, China
* Correspondence: ishadas2012@gmail.com

**This Supplementary file contains five tables (Tables S1 to S5)**

**Table S1.** The list of studies on air-water $CO_2$ and $CH_4$ fluxes, along with other related carbon-biogeochemistry parameters carried out in the Indian Sundarbans estuaries.

| Sl No. | Study Area | Time/Season of Sampling | Emphasis of the study | Reference |
|---|---|---|---|---|
| 1 | Sundarbans estuary (Saptamukhi, Thakuran, and Matla estuary) | April 2016 to January 2020 | Air-water $CO_2$ and $CH_4$ fluxes | 1 |
| 2 | Hooghly and Matla estuary | August 2018, January 2019 | Coupled Rn-222 and $pCO_2$ surveys | 2 |
| 3 | Dhanchi Island and Dimond Harbor | 27 January to 06 February 2018 | High temporal resolution direct measurement of $pCO_{2(water)}$ | 3 |
| 4 | Matla estuary | May 2017, August 2017, December 2017, and March 2018 | Seasonal $pCO_{2(water)}$ and air-water $CO_2$ fluxes | 4 |
| 5 | Hooghly-Sundarbans estuary | March to May 2016 | Pre-monsoon C biogeochemistry emphasizing on $pCO_{2(water)}$ and $CH_4$ dynamics | 5 |
| 6 | Hooghly estuary | June 2015 to May 2016 | Lateral flux of TAlk and DIC | 6 |
| 7 | Sadhupur, Pakhiralay, Dayapur, (Sundarban) | Winter, summer, pre-monsoon, monsoon (year not mentioned) | Seasonal fluctuation in greenhouse gas fluxes | 7 |
| 8 | Sundarbans estuary (Saptamukhi, Thakuran, and Matla estuary) | March 2017 | Diurnal variability and role of tides in governing C biogeochemistry | 8 |
| 9 | Hooghly-Sundarbans estuary | November 2017 | Post-monsoon C biogeochemistry | 9 |
| 10 | Hooghly-Sundarbans estuary | May and December 2014 | Lateral flux of DIC, DOC, and POC | 10 |
| 11 | Lothian Island and adjacent Saptamukhi estuary | 2010-2012 | Mangrove methane biogeochemistry | 11 |
| 12 | Hooghly and Matla estuary | August 2013 to July 2014 | Comparison of $pCO_{2(water)}$ and air-water $CO_2$ fluxes between river-dominated and mangrove-dominated marine estuary | 12 |
| 13 | Hooghly estuary and its adjacent coastal oceans | Winter and Summer of 2008 (January, February, May, September, November) | Estimation of $pCO_{2(water)}$ and air-water $CO_2$ fluxes using remote sensing techniques | 13 |
| 14 | Lothian Island and the adjacent Saptamukhi estuary | 2011-2012 | Exogenous $CH_4$ dynamics | 14 |
| 15 | Saptamukhi estuary | June (2010) to December (2011) | Photo-oxidation of $CH_4$ | 15 |
| 16 | Hooghly estuary, Lothian Island, Saptamukhi estuary | Pre-monsoon, 2014 | Biogeochemistry of organic matter | 16 |

| | | | | |
|---|---|---|---|---|
| 17 | Indian Sundarbans [Edwards Creek, Thakuran River, and Herobhanga River] | April and May 2011 | Spatial variability of $pCO_{2(water)}$ and air-water $CO_2$ fluxes in the pre-monsoon season | 17 |
| 18 | Saptamukhi estuary | June 2010 to December 2011 | $CH_4$ flux from porewater | 18 |
| 19 | Jharkhali mangroves and Hooghly estuary | December (2006) | Air-water $CH_4$ flux | 19 |
| 20 | Muriganga, Saptamukhi, and Thakuran rivers, Lothian Island, and Hooghly estuary (Diamond Harbour, Kachuberia, Beguakhali) | 2003 | Air-water $CH_4$ flux | 20 |
| 21 | Hooghly estuary | Every 2 weeks between 1999 and 2001 | Lateral fluxes of DIC and nutrients | 21 |
| 22 | Mooriganga, Saptamukhi, and Thakuran estuaries | January–December 2001 | Biogenic controls on the air-water $CO_2$ flux | 22 |
| 23 | Hooghly Estuary | January–December 1999 | Seasonal effects on Air-water $CO_2$ exchange | 23 |
| 24 | Mooriganga and Saptamukhi estuaries | August (1986) | Comparative analysis of $CO_2$ system in virgin and reclaimed mangrove waters | 24 |

**Table S2.** List of observations on TAlk and DIC from several studies on the Indian Sundarbans estuaries. The results are displayed either as mean ± standard deviation from the mean or as the range (minimum to maximum). NA denotes not available.

| Sampling Site/ Estuary | Time/ Season of Sampling (Year) | TAlk (μmol kg$^{-1}$) | DIC (μmol kg$^{-1}$) | Reference |
|---|---|---|---|---|
| Saptamukhi estuary | April 2016 to January 2020 | 1825 to 2506 | 1678 to 2203 | 1 |
| Thakuran estuary | April 2016 to January 2020 | 1711 to 2543 | 1641 to 2205 | 1 |
| Matla estuary | April 2016 to January 2020 | 1658 to 2380 | 1700 to 2108 | 1 |
| Hooghly estuary (surface water) | August 2018, January 2019 | 1720 to 2996 (2256±489) | 1671 to 3030 (2212±495) | 2 |
| Hooghly estuary (groundwater) | August 2018, January 2019 | 6136 to 18,178 (9087±5119) | 5671 to 16400 (8333±4547) | 2 |
| Matla estuary (surface water) | August 2018, January 2019 | 1735 to 2438 (2015±213) | 1938 to 2158 (1983±59) | 2 |
| Matla estuary (groundwater) | August 2018, January 2019 | 8099 to 9570 (8754±748) | 7664 to 9224 (8272±578) | 2 |
| Dhanchi Island and Dimond Harbor | 27th January to 6th February (2018) | 2100 to 2750 | 1900 to 2700 | 4 |
| Matla estuary | Pre-monsoon (2018) | 2243 to 2353 | 1995 to 2084 | 3 |

| | | | | |
|---|---|---|---|---|
| Matla estuary | Post-monsoon (2017) | 1757 to 1842 | 1730 to 1848 | 3 |
| Matla estuary | Monsoon (2017) | 1810 to 1915 | 1707 to 1783 | 3 |
| Sundarbans estuary (Saptamukhi, Thakuran, and Matla estuary) | March (2017) | 2190 to 2580 | 1920 to 2190 | 8 |
| Hooghly estuary (surface water) | November (2017) | 1797 to 2862 | 1678 to 2700 (2083±320) | 9 |
| Hooghly estuary (groundwater) | November (2017) | | 5655 to 11756 | 9 |
| Sundarbans estuary (surface water) | November (2017) | 2009 to 2289 | 1683 to 1920 (1756±73) | 9 |
| Sundarbans estuary (groundwater) | November (2017) | NA | 7524 to 13599 | 9 |
| Sundarbans estuary | March to May (2016) | 2350 to 2580 | 1690 to 1890 (1770±60) | 5 |
| Hooghly estuary | March to May (2016) | 2480 to 3110 | 1970 to 2680 (2270±220) | 5 |
| Hooghly estuary (Upper to lower estuary) | Monsoon (2015) | 2162 to 1499 | 1463 to 2167 | 6 |
| Hooghly estuary (Upper to lower estuary) | Pre-monsoon (2016) | 3676 to 2494 | 2460 to 3608 | 6 |
| Hooghly estuary (coastal area) | Annual (2015-16) | 1925 to 2455 | 1754 to 2167 | 6 |
| Lothian Island (mangrove) | Pre-monsoon (2014) | NA | 2220±40 | 10 |
| Hooghly estuary (upper estuary) | Pre-monsoon (2014) | NA | 2880±70 | 10 |
| Hooghly estuary (mid-estuary) | Pre-monsoon (2014) | NA | 2700±20 | 10 |
| Hooghly estuary (lower estuary) | Pre-monsoon (2014) | NA | 2400±30 | 10 |
| Lothian Island (mangrove) | Post-monsoon (2014) | NA | 2130±100 | 10 |
| Hooghly estuary (upper estuary) | Post-monsoon (2014) | NA | 2790±100 | 10 |
| Hooghly estuary (mid-estuary) | Post-monsoon (2014) | NA | 2570±20 | 10 |
| Hooghly estuary (lower estuary) | Post-monsoon (2014) | NA | 2210±40 | 10 |
| Hooghly estuary | Pre-monsoon (2014) | 2790 to 2970 | 2400 to 2880 | 16 |
| Saptamukhi estuary | Pre-monsoon (2014) | 2700 to 2760 | 2170 to 2260 | 16 |
| Hooghly estuary | August to July (2013-14) | 1615 to 5390 | 1559 to 5426 | 12 |
| Hooghly estuary and its adjacent coastal oceans | Winter (January, February, November) (2008) | NA | 1310±107<br>1711±19<br>1130±29 | 13 |
| Hooghly estuary and its adjacent coastal oceans | Summer (May and September) (2008) | NA | 990±27<br>1697±77 | 13 |

| | | | | |
|---|---|---|---|---|
| Jharkhali mangroves | December (2006) | 1820 to 2270 (2150±80) | 1910 to 2170 (2070±60) | 19 |
| Hooghly estuary | December (2006) | 2710 to 3210 (2910±180) | 2620 to 2930 (2770±110) | 19 |
| Mooriganga, Saptamukhi, and Thakuran estuaries | Monsoon (2001) | 1990±320 | NA | 22 |
| Mooriganga, Saptamukhi, and Thakuran estuaries | Post-monsoon (2001) | 1800±210 | NA | 22 |
| Mooriganga, Saptamukhi, and Thakuran estuaries | Pre-monsoon (2001) | 2700±350 | NA | 22 |
| Hooghly estuary | Monsoon (1999) | 1670±330 | NA | 23 |
| Hooghly estuary | Post-monsoon (1999) | 2200±430 | NA | 23 |
| Hooghly estuary | Pre-monsoon (1999) | 2590±270 | NA | 23 |

**Table S3.** List of observations on DOC and POC from several studies on the Indian Sundarbans estuaries. The results are displayed either as mean ± standard deviation from the mean or as the range (minimum to maximum). Single magnitudes, in some instances, represent the mean (without any reported standard deviation).

| Sampling Site/ Estuary | Time/ Season of Sampling (Year) | DOC (μmol $l^{-1}$) | POC (μmol $l^{-1}$) | Reference |
|---|---|---|---|---|
| Hooghly estuary (surface water) | Monsoon (2018) | 95.3 to 248.2 (182.1±47.1) | NA | 2 |
| Hooghly estuary (surface water) | Post-monsoon (2019) | 10.6 to 33.6 (22.8±7.9) | NA | 2 |
| Hooghly estuary (groundwater) | Monsoon (2018) | 51.9 to 83.4 (69.9±16.2) | NA | 2 |
| Hooghly estuary (groundwater) | Post-monsoon (2019) | 20.3 to 60.8 (40.5±28.6) | NA | 2 |
| Matla estuary (surface water) | Post-monsoon (2019) | 95.1 to 178.1 (145.0±30.7) | NA | 2 |
| Matla estuary (groundwater) | Monsoon (2018) | 124.4 to 190.6 (149.7±35.7) | NA | 2 |
| Matla estuary (groundwater) | Post-monsoon (2019) | 113.9 to 131.7 (125.6±10.1) | NA | 2 |
| Matla estuary | Pre-monsoon (2018) | 82.7 to 121.9 | 40.7 to 90.6 | 3 |
| Matla estuary | Post-monsoon (2017) | 105.7 to 170.1 | 24.2 to 83.9 | 3 |
| Matla estuary | Monsoon (2017) | 91.8 to 141.9 | 43.0 to 87.3 | 3 |
| Matla estuary | Pre-monsoon (2017) | 85.36 to 166.17 | 36.7 to 211.1 | 3 |
| Sundarbans estuary (Saptamukhi, Thakuran, and Matla estuary) | March (2017) | 109 to 287 | 130 to 508 | 8 |
| Hooghly estuary | November (2017) | 243 to 662 | 95 to 313 | 9 |
| Sundarbans estuary | November (2017) | 154 to 315 | 80 to 436 | 9 |

| | | | | |
|---|---|---|---|---|
| Hooghly estuary | March to May (2016) | 383 to 1068 | 138.6 to 699.9 | 9 |
| Sundarbans estuary | March to May (2016) | 358 to 808 | 37.49 to 91.29 | 9 |
| Lothian Island (mangrove) | Pre-monsoon (2014) | 294.3±34 | 28.0±8.6 | 10 |
| Hooghly River | Pre-monsoon (2014) | 205 | 49 | 10 |
| Hooghly estuary (upper estuary) | Pre-monsoon (2014) | 245.5±26.5 | 11.0±2.4 | 10 |
| Hooghly estuary (mid-estuary) | Pre-monsoon (2014) | 249.0±25.9 | 43.2±1.7 | 10 |
| Hooghly estuary (lower estuary) | Pre-monsoon (2014) | 324±27 | 27.5±3.6 | 10 |
| Lothian Island (mangrove) | Post-monsoon (2014) | 262.5±48.2 | 45.4±7.5 | 10 |
| Hooghly estuary (upper estuary) | Post-monsoon (2014) | 232.8±15.3 | 40.3±1.1 | 10 |
| Hooghly estuary (mid-estuary) | Post-monsoon (2014) | 263.6±26.7 | 129.7±6.7 | 10 |
| Hooghly estuary (lower estuary) | Post-monsoon (2014) | 223.5±31 | 57.5±3.2 | 10 |
| Hooghly estuary | Pre-monsoon (2014) | 245.4±26 to 324±27 | 11.04±2.5 to 28.9±1.66 | 16 |
| Saptamukhi estuary | Pre-monsoon (2014) | 260.3 to 328.2 | 27 to 37 | 16 |
| Jharkhali mangroves | December (2006) | NA | 8.8 to 47.9 (26.7±9.3) | 19 |
| Hooghly estuary | December (2006) | NA | 50 to 457 (183±109.0) | 19 |

**Table S4.** List of observations on $pCO_{2(water)}$ and air-water $CO_2$ fluxes from several studies on the Indian Sundarbans estuaries. The results are displayed either as mean ± standard deviation from the mean or as the range (minimum to maximum). Single magnitudes, in some instances, represent the mean (without any reported standard deviation).

| Sampling Site/ Estuary | Time/ Season of Sampling (Year) | $pCO_2$ (µatm) | Air-Water $CO_2$ Flux (µmol $m^{-2}$ $h^{-1}$) | Reference |
|---|---|---|---|---|
| Saptamukhi estuary | Annual (2016-20) | 180 to 2015 | -290 to 7230 | 1 |
| Thakuran estuary | Annual (2016-20) | 211 to 1867 | -860 to 4630 | 1 |
| Matla estuary | Annual (2016-20) | 439 to 2569 | -550 to 15110 | 1 |
| Hooghly estuary | Monsoon 2018 | 705 to 3679 (1873±9640) | 944 to 19024 | 2 |
| Hooghly estuary | Post-monsoon 2019 | 559 to 1711 (1090±481) | 265 to 4647 | 2 |
| Matla estuary | Monsoon 2018 | 464 to 1813 (1007±360) | 178 to 7584 | 2 |
| Matla estuary | Post-monsoon 2019 | 429 to 571 (508±33) | 35 to 513 | 2 |
| Creek (Dhanchi Island) | 27th January to 6th February (2018) | 315 to 1204 (470±162) | 13±34 to 107±284 | 4 |
| Estuary | 27th January to 6th February (2018) | 311 to 636 (387±58) | -4±12 to -37±95 | 4 |
| Matla estuary | Annual (2017-18) | 78 to 773 (327±195) | -100 to 2286 (983±644) | 3 |

| | | | | |
|---|---|---|---|---|
| Matla estuary | Pre-monsoon (2018) | 173 to 438 (297±79) | 702 to 1777 (1202±319) | 3 |
| Matla estuary | Post-monsoon (2017) | 78 to 386 (160±158) | -100 to 501 (208±205) | 3 |
| Matla estuary | Monsoon (2017) | 335 to 773 (523±126) | 981 to 2286 (1540±378) | 3 |
| Sundarbans estuary (Saptamukhi, Thakuran and Matla estuary) | March (2017) | 533 to 918 (690±129) | 398 to 3364 | 8 |
| Hooghly estuary | November (2017) | 267 to 4678 | -19.3 to 717 | 9 |
| Sundarbans estuary | November (2017) | 376 to 561 | -2.6 to 30.3 | 9 |
| Hooghly estuary | Pre-monsoon (2016) | 556 to 5002 | 2290 | 5 |
| Sundarbans estuary | Pre-monsoon (2016) | 268 to 418 | -162 | 5 |
| Hooghly estuary | 2015-16 | 1193±657 (475 – 3330) | 116 to 18759 | 6 |
| Lothian Island (mangrove) | Pre-monsoon (2014) | 634±112 | NA | 10 |
| Hooghly estuary (upper estuary) | Pre-monsoon (2014) | 992±60 | NA | 10 |
| Hooghly estuary (mid-estuary) | Pre-monsoon (2014) | 750±146 | NA | 10 |
| Hooghly estuary (lower estuary) | Pre-monsoon (2014) | 593±81 | NA | 10 |
| Lothian Island (mangrove) | Post-monsoon (2014) | 641±39 | NA | 10 |
| Hooghly estuary (upper estuary) | Post-monsoon (2014) | 441±13 | NA | 10 |
| Hooghly estuary (lower estuary) | Post-monsoon (2014) | 372±46 | NA | 10 |
| Hooghly estuary | Pre-monsoon (2014) | 592±81 to 1013±60 | NA | 16 |
| Saptamukhi estuary | Pre-monsoon (2014) | 506 to 712 | NA | 16 |
| Hooghly estuary | August to July (2013-14) | 2200 | 121 to 16944 | 12 |
| Matla estuary | August to July (2013-14) | 530 | -668 to 3033 | 12 |
| Herobhanga River | April and May (2011) | 507.98±73.69 | 28.1±13.24 | 17 |
| Thakuran River | April and May (2011) | 518.84±71.94 | 22.36±12.36 | 17 |
| Edwards Creek | April and May (2011) | 234.34±64.08 | -31.6±13.52 | 17 |
| Hooghly estuary (coastal waters) | Winter (January, February, November) (2008) | 320 to 500 | 57 | 13 |
| Hooghly estuary and its adjacent coastal oceans | Summer (May and September) (2008) | 450 | 10274 | 13 |
| Jharkhali mangroves | December (2006) | 353 to 476 (411±39.7) | 80 to 120 | 19 |
| Hooghly estuary | December (2006) | 930 to 1068 (996±40.5) | 670 to 1440 | 19 |

| | | | | |
|---|---|---|---|---|
| Mooriganga, Saptamukhi, and Thakuran estuaries | January–December (2001) | 416 to 1062 | -16.2 to 49.9 | 22 |
| Tip and base of Sagar Island and Diamond Harbour | January–December (1999) | 300 to 1200 (±200) | -2780 to 8440 | 23 |
| Mooriganga estuary | August (1986) | 830±363 | 967±421 | 24 |
| Saptamukhi estuary | August (1986) | 2210±1460 | 2363±1558 | 24 |

**Table S5.** List of observations on $CH_4$ concentration in water and air-water $CH_4$ fluxes from several studies on the Indian Sundarbans estuaries. The results are displayed either as mean ± standard deviation from the mean or as the range (minimum to maximum). Single magnitudes, in some instances, represent the mean (without any reported standard deviation).

| Sampling Site/ Estuary | Time/ Season of Sampling (Year) | $CH_4$ conc. in water (nmol $l^{-1}$) | Air-Water $CH_4$ Flux (µmol $m^{-2}$ $h^{-1}$) | Reference |
|---|---|---|---|---|
| Hooghly estuary | Pre-monsoon (2016) | 15.4 to 445.7 | 5.4 | 5 |
| Sadhupur mangrove | Winter | 83.8±1.2 to 652.7±8.8 | -8.3±0.4 to 2.9±0.2 | 7 |
| Sadhupur mangrove | Summer | 153.4±1.9 to 772.0±3.5 | -1.2±0.3 to 8.7±0.4 | 7 |
| Sadhupur mangrove | Pre-monsoon | 78.5±3.3 to 228.3±4.8 | 2.1±0.1 to 8.3±0.1 | 7 |
| Sadhupur mangrove | Monsoon | 85.7±5.6 to 1341.2±13.4 | 10.41±0.5 to 15.9±1.6 | 7 |
| Pakhiralaya mangrove | Winter | 104.8±3.2 to 159.0±3.5 | -4.6±0.6 to 5.8±0.1 | 7 |
| Pakhiralaya mangrove | Summer | 92.8±5.6 to 152.7±3.4 | -3.3±0.4 to 6.6±0.3 | 7 |
| Pakhiralaya mangrove | Pre-monsoon | 94.4±4.3 to 215.7±5.3 | 2.1±0.1 to 11.2±0.8 | 7 |
| Pakhiralaya mangrove | Monsoon | 71.7±3.6 to 1125.4±10.6 | 7.5±0.8 to 23.8±1.3 | 7 |
| Dayapur mangrove | Winter | 82.2±3.3 to 117.2±3.2 | -10.0±1.0 to 6.7±0.7 | 7 |
| Dayapur mangrove | Summer | 79.8±1.2 to 771.1±9.1 | -2.5±0.1 to 10.8±0.5 | 7 |
| Dayapur mangrove | Pre-monsoon | 94.4±4.4 to 149.3±3.9 | 5.9±0.4 to 9.2±0.2 | 7 |
| Dayapur mangrove | Monsoon | 83.8±4.3 to 327.2±7.8 | 14.2±1.4 to 16.7±0.9 | 7 |
| Lothian Island and adjacent Saptamukhi estuary | Pre-monsoon (2010-2012) | 47.28±12.85 to 54.20±5.06 | 0.3±0.1 | 11 |
| Lothian Island and adjacent Saptamukhi estuary | Monsoon (2010-2012) | 53.27±19.47 to 64.58±10.56 | 0.4±0.3 | 11 |
| Lothian Island and adjacent Saptamukhi estuary | Post-monsoon (2010-2012) | 67.97±33.12 to 90.91±21.20 | 0.4±0.1 | 11 |
| Saptamukhi estuary | June (2010) to December (2011) | 54.20±5.06 to 90.91±21.20 | 0.3 | 15 |

| | | | | |
|---|---|---|---|---|
| Saptamukhi estuary | Pre-monsoon (2010-2011) | 46.92±15.26 | 265.02±95.43 | 18 |
| Saptamukhi estuary | Monsoon (2010-2011) | 72.29±11.13 | 515.06±65.04 | 18 |
| Saptamukhi estuary | Post-monsoon (2010-2011) | 90.06±29.28 | 444.29±170.76 | 18 |
| Jharkhali mangroves | December (2006) | 5.8 to 30.9 (13.3±6.5) | 0.4 to 1.4 | 19 |
| Hooghly estuary | December (2006) | 3.6 to 29.8 (10.3±6.8) | 0.2 to 0.9 | 19 |
| Muriganga, Saptamukhi and Thakuran | Pre-monsoon (2003) | 42.31±20.96 | 0.30±0.16 | 20 |
| Muriganga, Saptamukhi and Thakuran | Monsoon (2003) | 35.75±15.48 | 0.45±0.38 | 20 |
| Muriganga, Saptamukhi and Thakuran (porewater) | Pre-monsoon (2003) | 814±572 | 12.97±55.84 | 20 |
| Muriganga, Saptamukhi and Thakuran (porewater) | Monsoon (2003) | 817±828 | 52.53±31.62 | 20 |
| Muriganga, Saptamukhi and Thakuran (porewater) | Post-monsoon (2003) | 1546±1562 | 48.00±67.53 | 20 |
| Hooghly estuary (Diamond Harbour) | Pre-monsoon (2003) | 54.39 to 59.25 | 371.1 to 2801.2 | 20 |
| Hooghly estuary (Kachuberia) | Pre-monsoon (2003) | 29.3 to 53.5 | 309.2 to 3658.4 | 20 |
| Hooghly estuary (Beguakhali) | Pre-monsoon (2003) | 42.45 to 46.88 | 5333.7 to 6193.5 | 20 |
| Hooghly estuary (Diamond Harbour) | Monsoon (2003) | 19.67 to 22.10 | 53.7 to 57.65 | 20 |
| Hooghly estuary (Kachuberia) | Monsoon (2003) | 17.25 to 23.62 | 61.6 to 107.9 | 20 |
| Hooghly estuary (Beguakhali) | Monsoon (2003) | 13.65 to 15.45 | 65.45 to 69.3 | 20 |
| Hooghly estuary (Diamond Harbour) | Post-monsoon (2003) | 23.25 to 45.33 | 39.93 to 1230.5 | 20 |
| Hooghly estuary (Kachuberia) | Post-monsoon (2003) | 18.44 to 49.23 | 99.1 to 375.52 | 20 |
| Hooghly estuary (Beguakhali) | Post-monsoon (2003) | 10.3 to 52.4 | 36.7 to 968.1 | 20 |

References


1. Acharya, A.; Sanyal, P.; Paul, M.; Gupta, V.K.; Bakshi, S.; Bhattacharyya, P.; Mukhopadhyay, S.K. Seasonal quantification of carbonate dissolution and $CO_2$ emission dynamics in the Indian Sundarbans estuaries. Reg. Stud. Mar. Sci. 2022, 53, 102413. https://doi.org/10.1016/j.rsma.2022.102413.
2. Akhand, A.; Chanda, A.; Watanabe, K.; Das, S.; Tokoro, T.; Hazra, S.; Kuwae, T. Drivers of inorganic carbon dynamics and air–water $CO_2$ fluxes in two large tropical estuaries: Insights from coupled radon ($^{222}Rn$) and $pCO_2$ surveys. Limnol. Oceanogr. 2022, 67, S118-S132, https://doi.org/10.1002/lno.12075.
3. Akhand, A.; Chanda, A.; Watanabe, K.; Das, S.; Tokoro, T.; Hazra, S.; Kuwae, T. Reduction in Riverine Freshwater Supply Changes Inorganic and Organic Carbon Dynamics and Air‐Water $CO_2$ Fluxes in a Tropical Mangrove Dominated Estuary. J. Geophys. Res. Biogeosci. 2021b, 126, https://doi.org/10.1029/2020JG006144.
4. Akhand, A.; Chanda, A.; Watanabe, K.; Das, S.; Tokoro, T.; Chakraborty, K.; Hazra, S.; Kuwae, T. Low $CO_2$ evasion rate from the mangrove-surrounding waters of the Sundarbans. Biogeochemistry. 2021a, 153, 95-114. https://doi.org/10.1007/s10533-021-00769-9.
5. Dutta, M.K.; Kumar, S.; Mukherjee, R.; Sharma, N.; Bhushan, R.; Sanyal, P.; Paul, M.; Mukhopadhyay, S.K. Carbon Bioge-ochemistry of Two Contrasting Tropical Estuarine Ecosystems During Premonsoon. Estuaries Coast. 2021, 44, 1916-1930, https://doi.org/10.1007/s12237-021-00908-3.
6. Ghosh, J.; Chakraborty, K.; Chanda, A.; Akhand, A.; Bhattacharya, T.; Das, S.; Das, I.; Hazra, S.; Choudhury, S.B.; Wells, M. Outwelling of total alkalinity and dissolved inorganic carbon from the Hooghly River to the adjacent coastal Bay of Bengal. Environ. Monit. Assess. 2021, 193, 1-14, https://doi.org/10.1007/s10661-021-09191-y.
7. Padhy, S.R.; Bhattacharyya, P.; Dash, P.K.; Reddy, C.S.; Chakraborty, A.; Pathak, H. Seasonal fluctuation in three mode of greenhouse gases emission in relation to soil labile carbon pools in degraded mangrove, Sundarban, India. Sci. Total Environ. 2020, 705, 135909. https://doi.org/10.1016/j.scitotenv.2019.135909.
8. Dutta, M.K.; Kumar, S.; Mukherjee, R.; Sharma, N.; Acharya, A.; Sanyal, P.; Bhusan, R.; Mukhopadhyay, S.K. Diurnal carbon dynamics in a mangrove-dominated tropical estuary (Sundarbans, India). Estuar. Coast. Shelf Sci. 2019b, 229, 106426. https://doi.org/10.1016/j.ecss.2019.106426.
9. Dutta, M.K.; Kumar, S.; Mukherjee, R.; Sanyal, P.; Mukhopadhyay, S.K. The post-monsoon carbon biogeochemistry of the Hooghly–Sundarbans estuarine system under different levels of anthropogenic impacts. Biogeosciences, 2019a, 16, 289-307, https://doi.org/10.5194/bg-16-289-2019.
10. Ray, R.; Baum, A.; Rixen, T.; Gleixner, G.; Jana, T. K. Exportation of dissolved (inorganic and organic) and particulate carbon from mangroves and its implication to the carbon budget in the Indian Sundarbans. Sci. Total Environ. 2018, 621, 535-547, https://doi.org/10.1016/j.scitotenv.2017.11.225.
11. Dutta, M.K.; Bianchi, T.S.; Mukhopadhyay, S.K. Mangrove methane biogeochemistry in the Indian Sundarbans: a proposed budget. Front. Mar. Sci. 2017, 4, 187, https://doi.org/10.3389/fmars.2017.00187.
12. Akhand, A.; Chanda, A.; Manna, S.; Das, S.; Hazra, S.; Roy, R.; Choudhury, S.B.; Rao, K.H.; Dadhwal, V.K.; Chakraborty, K.; Mostofa, K.M.G. A comparison of $CO_2$ dynamics and air‐water fluxes in a river‐dominated estuary and a mangrove‐dominated marine estuary. Geophys. Res. Lett. 2016, 43, 11-726. https://doi.org/10.1002/2016GL070716.
13. Padhy, P.C.; Nayak, R.K.; Dadhwal, V.K.; Salim, M.; Mitra, D.; Chaudhury, S.B.; Rao, P.R.; Rao, K.H.; Dutt, C.B.S. Estimation of partial pressure of carbon dioxide and air-sea fluxes in Hooghly

estuary based on in situ and satellite observations. J. Indian Soc. Remote Sens. 2016, 44, 135-143, https://doi.org/10.1007/s12524-015-0459-z.
14. Dutta, M.K.; Mukherjee, R.; Jana, T.K.; Mukhopadhyay, S.K. Biogeochemical dynamics of exogenous methane in an estuary associated to a mangrove biosphere; the Sundarbans, NE coast of India. Mar. Chem. 2015a, 170, 1-10. https://doi.org/10.1016/j.marchem.2014.12.006.
15. Dutta, M.K.; Ray, R.; Mukherjee, R.; Jana, T.K.; Mukhopadhyay, S.K. Atmospheric fluxes and photo-oxidation of methane in the mangrove environment of the Sundarbans, NE coast of India; A case study from Lothian Island. Agric. For. Meteorol. 2015b, 213, 33-41. https://doi.org/10.1016/j.agrformet.2015.06.010.
16. Ray, R.; Rixen, T.; Baum, A.; Malik, A.; Gleixner, G.; Jana, T.K. Distribution, sources and biogeochemistry of organic matter in a mangrove dominated estuarine system (Indian Sundarbans) during the pre-monsoon. Estuar. Coast. Shelf Sci. 2015, 167, pp.404-413. https://doi.org/10.1016/j.ecss.2015.10.017.
17. Akhand, A.; Chanda, A.; Dutta, S.; Manna, S.; Sanyal, P.; Hazra, S.; Rao, K.H.; Dadhwal, V.K. Dual character of Sundarban estuary as a source and sink of $CO_2$ during summer: an investigation of spatial dynamics. Environ. Monit. Assess. 2013, 185, 6505-6515, https://doi.org/10.1007/s10661-012-3042-x.
18. Dutta, M.K.; Chowdhury, C.; Jana, T.K.; Mukhopadhyay, S.K. Dynamics and exchange fluxes of methane in the estuarine mangrove environment of the Sundarbans, NE coast of India. Atmos. Environ. 2013, 77, 631-639, https://doi.org/10.1016/j.atmosenv.2013.05.050.
19. Neetha, V. Dissolved carbon dioxide and methane in estuaries and waters surrounding mangroves on the East coast of India and the Andaman Islands. Ph.D, Anna University, Chennai, October, 2008. Available online: http://hdl.handle.net/10603/29692 (accessed on 17.10.2022).
20. Biswas, H.; Mukhopadhyay, S.K.; Sen, S.; Jana, T.K. Spatial and temporal patterns of methane dynamics in the tropical mangrove dominated estuary, NE coast of Bay of Bengal, India. J. Mar. Syst. 2007, 68, 55-64, https://doi.org/10.1016/j.jmarsys.2006.11.001.
21. Mukhopadhyay, S. K.; Biswas, H. D. T. K.; De, T. K.; Jana, T. K. Fluxes of nutrients from the tropical River Hooghly at the land–ocean boundary of Sundarbans, NE Coast of Bay of Bengal, India. J. Mar. Syst. 2006, 62, 9-21, https://doi.org/10.1016/j.jmarsys.2006.03.004.
22. Biswas, H.; Mukhopadhyay, S.K.; De, T.K.; Sen, S.; Jana, T.K. Biogenic controls on the air—water carbon dioxide exchange in the Sundarban mangrove environment, northeast coast of Bay of Bengal, India. Limnol. Oceanogr. 2004, 49, 95-101. https://doi.org/10.4319/lo.2004.49.1.0095.
23. Mukhopadhyay, S.K.; Biswas, H.; De, T.K.; Sen, S.; Jana, T.K. Seasonal effects on the air–water carbon dioxide exchange in the Hooghly estuary, NE coast of Bay of Bengal, India. Environ. Monit. Assess. 2002, 4, 549-552. DOI https://doi.org/10.1039/B201614A.
24. Ghosh, S.; Jana, T.K.; Singh, B.N.; Choudhury, A. Comparative study of carbon dioxide system in virgin and reclaimed mangrove waters of Sundarbans. Mahasagar, 1987, 20, 155-161, http://www.ijs.nio.org/index.php/msagar/article/view/2135.
